# Cervical cancer in Zimbabwe: a situation analysis

**DOI:** 10.11604/pamj.2017.27.215.12994

**Published:** 2017-07-21

**Authors:** Oppah Kuguyo, Alice Matimba, Nomsa Tsikai, Thulani Magwali, Mugove Madziyire, Muchabayiwa Gidiri, Collet Dandara, Charles Nhachi

**Affiliations:** 1 University of Zimbabwe College of Health Sciences, Department of Clinical Pharmacology, Avondale, Harare, Zimbabwe; 2 University of Zimbabwe College of Health Sciences, Radiology Department, Harare, Zimbabwe; 3 University of Zimbabwe College of Health Sciences, Department of Obstetrics and Gynaecology, Avondale, Harare, Zimbabwe; 4 University of Cape Town, Department of Pathology and Institute of Infectious Disease and Molecular Medicine, Division of Human Genetics, Faculty of Health Sciences, Observatory 7925, Cape Town, South Africa

**Keywords:** cervical cancer, screening, treatment, HPV testing, pap smear, VIAC, Zimbabwe, LMIC, Sub-Saharan Africa, HPV vaccine

## Abstract

**Introduction:**

Despite the wide-spread availability of cervical cancer prevention and screening programs in developed countries, the morbidity and mortality rates of cervical cancer in Zimbabwe are still very high. Limited resources as well as the high HIV prevalence are contributors to the high burden of cervical cancer. This paper aims to analyse the policies, frameworks and current practices in the management of cervical cancer in Zimbabwe.

**Methods:**

A review of national documents and published literature on cervical cancer prevention, screening, treatment and knowledge in Zimbabwe was done. Informal interviews were conducted to assess the practices of cervical cancer management.

**Results:**

Through strategic collaboration, a pilot for the HPV vaccination program is underway. The VIAC national cervical cancer screening program is being adopted into the current healthcare system. With regards to the treatment of precancerous lesions we found that the "see and treat" program has been implemented in colposcopy clinics. In addition, there are two multidisciplinary cancer treatment clinics installed in two central public hospitals. The general knowledge and understanding of cervical cancer is poor in Zimbabwe.

**Conclusion:**

Limitations in resources, infrastructure, manpower, delays in treatment and patient knowledge play a role in the high morbidity and mortality of cervical cancer in Zimbabwe. The Ministry of Health needs to increase funding to expedite the availability of HPV vaccine and screening programs. Community engagement initiatives to raise awareness on cervical cancer should be established to provide education on how to prevent the development of cervical cancer, as well as promote screening for early detection.

## Introduction

Cervical cancer is the fourth most commonly diagnosed cancer among females worldwide [1]. In 2012, the International Agency for Research on Cancer (IARC) recorded 527,624 new cervical cancer cases and 265,672 related deaths [2]. An estimated 90% of the globally recorded cervical cancer-related deaths are in low-and middle-income countries (LMICs), for which 8 in 10 are recorded within the Sub-Saharan African region [3, 4]. The morbidity and mortality of cervical cancer is much lower in developed countries due to availability of efficient and accessible screening programs as well as diagnostic and treatment facilities [5]. Meanwhile, in LMICs where the bulk of global cervical cancer cases are diagnosed (>85%) there is a poor survival rate attributable to late presentation at diagnosis and patients not receiving or completing their prescribed treatment regimens [6]. In LMICs there are challenges in affordability and availability of drugs, as well as access to treatment facilities [7-8]. The biggest risk factor of cervical cancer is human papillomavirus (HPV), commonly detected in cervical tumour specimens. The sexually transmitted infection, genital warts that are caused by high-risk HPV subtypes, have been shown to present with a 99% chance of progressing to cervical cancer [9]. Administering broad-spectrum HPV vaccines to adolescents in developed countries such as France, Iceland, Norway, Switzerland, UK and USA has decreased the prevalence of cervical cancer, thus proving successful as a preventive measure [10].

Global trends show that most cervical cancer high-risk countries are in Africa especially Malawi, Mozambique, Zambia and Zimbabwe [11]. Similar to worldwide statistics, cervical cancer is the most frequently occurring cancer in women of all races and ages in Zimbabwe, with a burden of 19% [12]. In black women cervical cancer contributes to 35.5% of all cancers while in non-black women it accounts for 2.8% [12]. It is estimated that 2270 women are diagnosed with cervical cancer in Zimbabwe annually and a mortality rate of 64% has been recorded [13]. The burden and mortality rate of cervical cancer is most likely to be higher than those recorded in the national cancer registry because some cases go unreported in areas that have poor access to health facilities such as rural areas [14]. The burden of cervical cancer is still very high in Zimbabwe mainly as a result of late presentation of disease, poor screening, diagnosis and treatment facilities which is compounded by the very high HIV incidence. In 2015, 1.4 million people were estimated to be living with HIV in Zimbabwe, and HIV augments the risk of malignancy by 10% [15, 16]. There is a host of articles to address and report on prevention, screening and treatment in biomedical, behavioural and policy level findings of cervical cancer that have been published in different settings. The aim of this paper is to conduct a situation analysis on cervical cancer in Zimbabwe by reviewing the policy frameworks and practices in prevention, early detection and treatment of cervical cancer. This paper will highlight successes and challenges in the prevention and progressive management of cervical cancer in Zimbabwe.

## Methods

**Figure 1:**
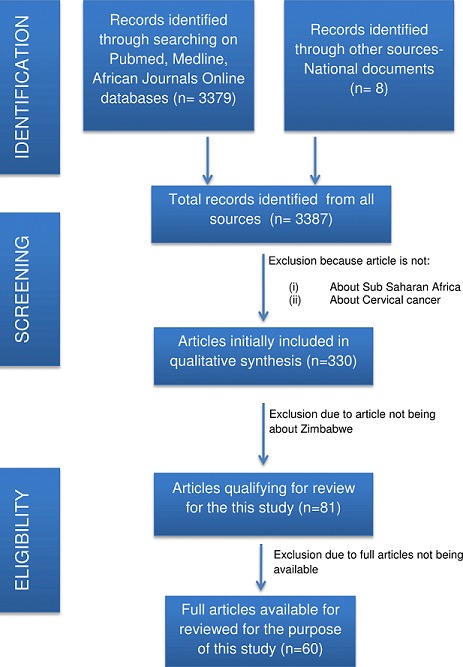
PRISMA flow diagram of the articles searches yielded, excluded and reviewed for the purpose of this study

A review of national documents was done by conducting searches in the Zimbabwe Ministry of Health and Child Care and Zimbabwe National Cancer Registry databases. Reference documents reviewed were: National Cancer Strategy of Zimbabwe (2013-2017); Five annual reports between 2010 and 2014 of Zimbabwe National Cancer Registry; ICO Information Centre on Human Papillomavirus and cancer: Zimbabwe document (2017) [17]. Secondly, a literature search on PubMed/Medline (NCBI), Google Scholar and African Journals Online databases was conducted. The searches yielded are shown in Figure 1. A total of 3387 articles were retrieved and 330 were about cervical cancer prevention, screening and treatment in Sub-Saharan Africa. Duplicates were eliminated. Emphasis was made on publications that were focussing on Zimbabwe (81 publications), for which 60 full articles were available for review. The inclusion criteria for articles to be used for this study were the cervical cancer-related publications about Zimbabwe. Searches in the databases used key terms "Cervical cancer + Zimbabwe"; "Cervical Cancer + sub-Saharan Africa"; "Cervical cancer + Africa"; "HPV vaccine in Zimbabwe"; "VIAC + Zimbabwe"; "Screening + Cervical cancer". To access information about the current practices of cervical cancer prevention, screening and treatment, Parirenyatwa Group of Hospitals (PGH) Radiotherapy and Chemotherapy Centre, PGH Colposcopy clinic, and Harare Central Hospitals Spillhaus Colposcopy Clinic were visited for informal interviews with health care providers. Furthermore, consultations with surgeons, gynaecologists and oncologists from the private and public sectors were done (Table 1).

**Table 1:** The professions and questions asked in informal interviews

Profession	Participants	Questions asked
Nurse	8	1. What is the availability of pap smear for the general public?
		2. How much do Pap smears cost in your institution?
General practitioners	4	3. Does one need to be referred for a pap smear?
		4. What is the cervical cancer screening method used in your institution?
Oncologists	3	5. Who conducts pap smears? Gynaecologist or nurse?
		6. Who conducts VIACs in your institution?
Gynaecologists	5	7. Where does one get referred to upon detection of irregularities?

* Nurses were recruited from Parirenyatwa (n=4) and Harare Central Hospitals (n=4)

## Results

**Figure 2:**
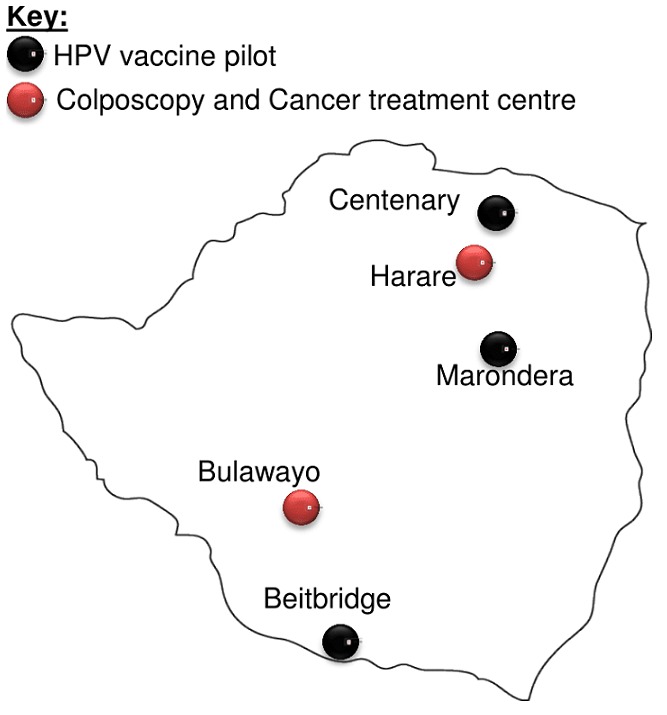
A geographical representation of where HPV vaccine is being run as a pilot and sites with the treatment centres for pre-cancerous and cancerous lesions

Findings of studies published about cervical cancer in Zimbabwe are summarised in Table 2. There is no national HPV vaccination programme to prevent cervical cancer, however, there are two pilot programmes being run in three different towns Marondera, Beitbridge and Centenary (Figure 2). A Vaccine Alliance (GAVI), UNICEF, UNFPA, WHO and Zimbabwe Expanded Programme of Immunisation (ZEPI) collaboration introduced the HPV vaccine in Marondera and Beitbridge in 2014 using a school-based and community-based strategy to access 10-year-old girls in and out of school. An additional program sponsored by the Roman Catholic Church Missionary is currently underway in Centenary at St Albert's Mission Hospital. HPV testing is offered at Lancet laboratories and is not accessible to all individuals due to high cost. The national pap smear screening program was stopped due to limitations in manpower and infrastructure. Another resource offering pap smears is the National Family Planning Council, subject to availability of resources, while private gynaecologists offer the same service at an approximate 7-fold of the cost (Table 3). In some cases, medical aid societies subsidise up to 50% of such costs leaving the patient to cover the short-fall. VIAC is the current national cervical cancer screening program. VIACs are available in all public hospitals and some clinics.

**Table 2:** Findings from the literature search on cervical cancer-related prevention, screening and treatment methods in Zimbabwe

	Outcome
**Primary Prevention**	No national screening or HPV testing programmes
	Two pilot programs are in place in Marondera, Beitbridge and Centenary
	UNICEF intends to scale up the pilot to the rest of the country in 2016
**Screening**	No consistent cervical cancer screening in Zimbabwe
	Unsuccessful national pap smear program
	Current adaptation of VIAC national screening program
	Of 514 participants, 91% had not been screened for cervical cancer; 81% did not know about cervical cancer screening; 80 % were interested in screening after education intervention
	HPV DNA testing was examined as a mode of screening however low specificity thus need for co-screening.
**Treatment**	Single visit approach of VIAC followed by LEEP or cryotherapy is feasible and safe
	Most cervical cancer patients present late, not qualifying for surgery or radiotherapy
	Chemoradiation is most common form of treatment administered
**Knowledge, Perception and attitudes**	Cervical cancer is the dirtiness of the womb caused by sperm retention
	Causes of cervical cancer- vaginal preparations (vaginal herb/ chemical use), multiple sexual partners, cold weather and witchcraft,
	Poor understanding of predisposing factors of cervical cancer and screening No knowledge of HPV and vaccines

**Table 3:** Costs of cervical cancer preventive and screening/early detection methods in the public hospitals compared to in the private hospitals

	Cost in Public sect (USD)	Cost in Private sect (USD)	
**Prevention**	HPV vaccine	Not available	180-300
**Screening**	Pap Smear	20*	150
	VIAC	Free*	10-20

* = subject to availability

Treatment is offered at public colposcopy clinics and radiotherapy and chemotherapy centres located in central hospitals in two cities in Zimbabwe, Harare (PGH) and Bulawayo (Mpilo hospital), illustrated in Figure 2. The screen and treat method using LEEP and cryotherapy has been piloted at Mpilo Hospital and is feasible for identifying and treating cervical intraepithelial lesions. There are no pharmaceutical companies in Zimbabwe that produce chemotherapy drugs, such as cisplatin thus causing delays in treatment due to delayed delivery. For patients that are terminally ill, hospice services are available at low costs or free of charge such as the Island Hospice and Health and Hospice and Palliative Care Association of Zimbabwe (HOSPAZ). The healthcare professionals reported that there are no pap smears in the public hospitals, while private hospitals and the national family planning clinics offer them at a cost shown in Table 3. A referral letter is not required for a pap smear. VIACs are the screening method offered in all public health care for free and are conducted by nurses. Suspicious VIAC results warrant referral to the Spillhaus Colposcopy Clinic at Harare Central Hospitals or Parirenyatwa Group of Hospitals Colposcopy Clinic in Harare or alternatively Mpilo Central Hospital Colposcopy Clinic in Bulawayo.

## Discussion

**Prevention**: The most effective preventive strategies intercept the initial offset of cervical cancer by inhibiting the activity of the causative agent [17-19]. HPV has been implicated as the main aetiological factor in 99% of cervical cancer cases [20]. Seven HPV subtypes account for 87% of cervical cancer and HPV16/18 collectively contribute to 70% [21]. Seventy out of one hundred and ninety-six countries implemented HPV vaccinations as national programs [22]. Zimbabwe is one of the countries that are still in the pilot phase for a three-dose schedule for Cervarix (HPV16/18) and Ordasit (HPV6/11/16/18) HPV vaccine programme [23]. Over 35% of women contract HPV within two years of on-setting sexual activity so this pilot in Zimbabwe targets 10 year old girls or girls that have not lost their virginity [24-27]. The vaccine is safe to administer to immunocompromised individuals [28]. It is hoped that these vaccination programs will lead to a decrease in cervical cancer which can then incentivise the Ministry of Health to mobilise resources for national implementation. Since the national HPV vaccine programme in Australia, the incidence of genital warts has significantly decreased in women < 27years [29]. Correspondingly, cervical cancer has decreased in USA, UK, Switzerland and Austria since implementing the national vaccination programme [30-33]. South Africa and Botswana [34, 35] adopted the national vaccination program in 2015, so impact is not yet reported. The prevalent HPV subtypes in Zimbabwe are HPV 16, 18, 31, 33, 35, 45, 51, 52 and 56 and can co-vitiate [13]. The vaccines being piloted in Zimbabwe target only four HPV subtypes 6, 11, 16 and 18 for which only two intersect with ones observed in Zimbabwe. These vaccines may not be sufficient to prevent cervical cancer pathogenesis in a setting with high HPV diversity. However, HPV vaccines show anamnestic cross-protection against other oncogenic HPV subtypes by identifying the capsid proteins and triggering a broad immune response [36, 37]. While there is a possibility of cross-protection, it is not guaranteed to prevent pathogenesis of the HPV subtypes that are not targeted by these vaccines. Additionally, 17% of HPV-related cancers are caused by two or more HPV subtypes, thus cross-protection may unlikely be dependable [38]. The nanovalent vaccine to target HPV6, 11, 16, 18, 31, 33, 45, 52 and 58 was approved targeting five HPV types that are prevalent in Zimbabwe, thus may be more beneficial compared to the available vaccines [39, 40].

HPV vaccines are expensive and available in private healthcare in Zimbabwe (Table 3). Majority of Zimbabweans are informally employed, of low socioeconomic status and rely heavily on the erratic public healthcare. Lacking national vaccination programmes in the public health sector socioeconomically discriminates against the public health sector clientele by limiting the knowledge of and accessibility to such services. Therefore, in order to make vaccines available to all women, regardless of socioeconomic status, the vaccination programs need to expand into the public sector, at a lower price through subsidiary funding from the government or donors. With provision of subsidiary funds and political motivation, an intensive vaccination campaign model can be adopted from neighbouring LMICs such as Botswana. The vaccination was carried out bi-annually, in country-wide clinics and schools on set dates (over a week period) in Botswana, ensuring a wide coverage in limited time [34].

Knowledge about cervical cancer is varied among Zimbabweans, with some women from rural areas describing cervical cancer as" *a dirtiness of the womb caused by sperm" and some further alluding to the cause as; "vaginal preparations, multiple sexual partners, cold weather and witchcraft*" (Table 2) [41]. This poor understanding along with the misconceptions of what cancer and cervical cancer is, leads to poor health seeking behaviour. Therefore, it is important to conduct education and awareness campaigns to improve the knowledge, attitudes and perceptions of cervical cancer. This is supported by findings that after education on cervical cancer, Taiwanese women were interested in HPV vaccination programs, under the condition of personal discretion [42]. Similarly Nigerian women, after education on HPV reported they would recommend the vaccine to their female children [43]. It is very common in conservative cultures not to address issues that concern reproductive health. Furthermore, because the HPV vaccine targets minors, assent is required before vaccination therefore reproductive health education should be conducted at community and school levels for better sociocultural acceptance. Health issues among Zimbabweans traditionally are not seen as individual concerns, but as familial or community challenges. Thus acceptability at family and community level is a primer for success of any health initiatives.

Screening: Early detection can be achieved by conducting regular screening [44]. WHO proposes screening to identify asymptomatic precancerous lesions such as cervical intraepithelial neoplasia (CIN) between 30-49 years regardless of HPV vaccination status [45]. Several cervical cancer screening methods can be used, and can be classed into cytological tests-papanicolau smear test (Pap smear); liquid based cytology; visual inspection tests-acetic acid (VIAC) and Lugol's iodine (VILI); and HPV DNA testing [46]. Pap smear is one of the most reliable and effective screening methods. In developed countries, national pap smear screening programs have significantly decreased the burden of cervical cancer [47] while LMICs have been unsuccessful in implementing such programs [34, 48]. In particular, administering pap smear as routine care has decreased the incidence of cervical cancer in UK, Switzerland, USA and Australia [31, 49]. However, pap smears were not easily adopted into routine care in the public health sector in Zimbabwe due to limitations in resources and lack of skilled cytopathologists and cytotechnologists to conduct the smear analysis [50].

The use of contraceptives induces cervical inflammation resulting in CIN, a precursor to cervical cancer [51-53]. In Zimbabwe pap smears were routine care for women on the injection contraceptive Depo-Provera, from 1987 until late 1990s but was halted due to inadequate resources [54]. In addition other primary care facilities and district hospitals previously offered pap smears to women six weeks post-delivery, which was terminated due to lack of relevant infrastructure and resources [55]. These challenges resonate with challenges in other LMICs like Nigeria, India and Ghana causing non-screening using pap smears [56]. In 2017, pap smears are still offered in Zimbabwe at family planning clinics subject to availability of consumables or through specialists and gynaecologists at costs indicated in Table 3. Additionally, medical aid societies subsidise the cost of pap smears however, this has low impact on prevention because the number of individuals subscribed to medical aid is very low due to financial constraints. It is recommended that between 21-65 years, women undergo cytological testing every three years and HPV co-testing every five years after 30 years [57]. HPV co-testing increases the chance for HPV detection and CIN diagnosis by 30% [14].

This could be done in Zimbabwe with the regular service provided by one private laboratory, Lancet, however these tests are expensive and beyond reach for the majority who need them. In light of the cost of pap smears and HPV DNA testing versus the country's economic standing, the cumulative cost of co-testing is very high for the average Zimbabwean woman. In addition to costs incurred by patients, studies have also highlighted that in LMICs only about two thirds of women are interested in and follow up their pap smear results [58-60]. Since pap smears are not point of care tests and the follow-up for results can be very poor, it would be very difficult to advocate for resource-constrained governments to subsidise funds for these tests. However, through public participation, an interest to follow up on results can be enhanced as a first step in the awareness campaign. The VIAC is the most commonly used screening method in Zimbabwe [61]. It has a fast turnaround time of approximately five minutes and is an easy method that can be carried out by nurses [62]. In the private sector VIACs are affordable (Table 3), while in public hospitals they are free. VIACs are therefore a favourable alternative in a setting that has technical, infrastructure and financial limitations [56]. VIAC has higher sensitivity than the conventional pap smear and so is more successful in settings were rescreening is unlikely [63-65]. In addition, VIACs do not require second opinions for result interpretation or secondary visits for result collection [66]. In India, a similar LMIC, a single round of VIAC decreased the morbidity and mortality of cervical cancer by 25% and 35% respectively after a 7 year period [67]. Moreover, because VIAC has a rapid turnaround time, treatment can be conducted in the same visit in the "screen and treat" method devised by the Alliance for cervical cancer prevention [68, 69]. The screen and treat programme has been shown as a safe and feasible way of detecting precancerous lesions, when implemented in countries such as Botswana, India, Thailand, Bangladesh, Brazil and Mexico [34, 70-72]. Furthermore, the screen and treat method has been adopted as point of care in developed countries such as China, Latin Americas and UK [73]. In Zimbabwe VIACs are offered at all tertiary, provincial, most district hospitals and small clinics. In LMICs, resource and medical manpower can sometimes lead to inadequate screening, thus in such settings training general healthcare providers has helped in decentralizing VIAC screening. Fear of finding out health status has been shown to be one of the barriers hindering screening and testing [74]. Enlightenment about the importance of early detection is key in improving the perceptions of cervical cancer. Creating awareness on the importance of screening and where such services are available would increase the cervical cancer screening prevalence.

**Treatment**: The treatment of precancerous cervical lesions such as CIN is by referral to Colposcopy and gynaecology clinics located at Harare central hospital, PGH Colposcopy clinic and Mpilo Central Hospital (**Figure 2**). Here patients are treated using cryotherapy and loop electrosurgical excision procedure (LEEP). Overall cure rate of LEEP (96.4%) is higher compared to cryotherapy (88.3%), therefore both are viable treatment options for low-/high-grade CIN [75]. Cryotherapy effectively prevents future development of cervical disease, even in HPV-positive women therefore, it is recommended for screened HPV-positive women without confirmation of disease [76]. However in resource-scarce settings such as rural areas cryotherapy is favoured over LEEP. The success of LEEP depends on various factors, however HIV-positive women have an 86% success rate versus 100% in HIV negative women [77]. A challenge faced with the use of LEEP is overtreatment as a result of difficulty in classifying CIN, which may result in reproductive health challenges [78]. Therefore to avoid overtreatment, it is recommended that LEEP especially in the "screen and treat" is administered by experienced colposcopists with the ability to accurately classify cervical intraepithelial neoplasia and prescribe effective doses. The colposcopy clinics in Zimbabwe are equipped with resources to treat precancerous lesions however there is a challenge with shortage of manpower. In some instances there are a lot of patients that need attention, overwhelming the healthcare workers and resulting in a slow service. Should the initiative to advocate for screening be pushed, the volumes at the colposcopy clinics would increase and this would demand more manpower as well as more colposcopy clinics around the country. Advanced CIN can be managed surgically in women past child bearing age and with co-morbidities such as uterine fibroids. A common surgical approach, hysterectomy is used for treatment. Early stage cervical cancer, is treated using a hysterectomy, removal of the cervix and surrounding lymph nodes. The treatment of invasive cervical cancer ranges from radiotherapy and chemotherapy-which are administered dependent on tumour staging, patient performance status and age. As cervical cancer progresses, treatment is chemoradiation for curative purposes. Due to lack of screening, most women in Zimbabwe are diagnosed with cervical cancer at advanced stage (IIb-IIIb) which has a prognosis of 63% 5-year survival rate, 57% remission rate with treatment and 42% recurrence [79-81]. Therefore the treatment administered is chemoradiation using cisplatin. There are various private cancer care centres around the country, however most patients seek treatment in the public sector at the two state-owned facilities, the PGH Radiotherapy and Chemotherapy Centre and Mpilo Central Hospital (Figure 2). The cancer treatment centres are equipped with simulators that can be used for 3D radiotherapy [82].

While this machinery provides for improved therapy, it also requires rigorous and frequent maintenance by skilled technicians. A major limitation experienced in state-owned facilities is maintaining equipment, infrastructure and consumables stock resulting in inconsistent service delivery. Local pharmaceutical companies do not manufacture chemotherapy drugs such as cisplatin [82]. As a result, access to these drugs can be erratic at times. Thus the lack of availability of drugs can decrease chances of successful therapy. Furthermore the general rate of compliance to prescribed medication and regimes is low due to non-adherence by some patients caused by financial hardships, religious or cultural beliefs. Given the high incidence of cervical cancer and associated mortality in Zimbabwe, there is need to increase specialists such as nurses, radiographers, medical biophysicists and oncologists. This is not going to happen overnight because Zimbabwe is a low income country thus continues to lose specialists (brain-drain) due to low levels of remuneration when compared to compatriots in other parts of the world. In addition, public cancer treatment centres for the whole of Zimbabwe, are found only in two cities, meaning that many of the patients need to travel long distances to access care. More so, anticancer therapy requires frequent hospital visits which can span from weeks to months for complete treatment. So transportation for patients to travel is also a major factor that affects compliance to treatment and treatment outcomes. Anticancer therapy results in varying treatment outcomes between patients. Significant inter-patient variations in treatment outcomes can be attributed to genetic variations in the host or tumour. The component of tumour response that is affected by tumour and host genetics is studied in an emerging area, pharmacogenomics. However there is very little genomics research in Zimbabwe to understand the pharmacogenomics of anticancer drug responses. Genomic markers have the potential of identifying in individual patients, profiles that can determine their prognosis on particular cancer medications. It is hoped that increased knowledge in pharmacogenomics will lead to decreases in adverse drug responses in patients under treatment. In addition to therapy aimed at curing the disease, late stage patients receive palliative care. There are hospice services in Zimbabwe that offer support for cancer patients under palliative regimens. Examples of such facilities include the Hospice and Palliative Care Association of Zimbabwe (HOSPAZ) and Island Hospice and Healthcare. These hospices offer non-fee paying palliative care in-hospital or at home. At these hospices, families are taught to manage and care for cancer patients, and as well provide support to the affected families by offering counselling [**83**]. While these hospices are available, knowledge concerning the role of these hospices is very minimal and should be made more available to those in need. This includes the use of media to advertise, as well as more assertive use of social media to disseminate the importance of having such support structures.

## Conclusion

Cervical cancer is one of the deadliest diseases in low income countries such as Zimbabwe, even though it is preventable. Routine screening and early treatment can prevent 8 in 10 cervical cancer cases, if the abnormalities in the cervix are identified early. HPV vaccines hold a promise in the fight against cervical cancer and there are already calls for more genetically diverse vaccines to be used so as to cover regions affected by many different HPV subtypes. With the increased diversity of HPV subtypes detected in Zimbabwe, it would be of great benefit to access the nanovalent HPV vaccine as a potential preventive measure. Community engagement is an important activity that should be undertaken to improve the uptake of health initiatives by communities including early screening for cervical cancer. The main challenges faced in preventing, screening and treating cervical cancer are lack of human and material resources. Moreover, another challenge is the centralisation of facilities to vaccinate, screen and treat cancer therefore decentralisation of services would make them more accessible. Governments should take seriously the severity of the threat cervical cancer poses and allocate adequate resources and funds towards the prevention, screening and treatment of cervical cancer. This can be done through subsidizing costs for screening and treatment, so as to make them affordable and available in the public health sector. Use of genomics especially pharmacogenomics should be explored in efforts to contribute to precision medicine. Moreover, personalizing therapy is also very economical in that patients only receive therapy they would benefit from. Furthermore, implementing educational and awareness campaigns across the country with a primary focus on high risk areas such as rural areas and high risk women such as sex workers should cultivate a health seeking behavior. While developing these educational tools there is a growing need to address cultural and socioeconomic factors that mitigate public health awareness.

### What is known about this topic

- Cervical cancer is a preventable disease. The burden has been decreased by providing free access to preventive measures such HPV vaccine and screening.

### What this study adds

- This study highlights the gaps in the cervical cancer management in Zimbabwe;
- There is need for financial resources to be mobilized towards increasing the accessibility of cervical cancer prevention and screening;
- The cancer treatment facilities need to be decentralized into hospitals across the country for better coverage.

## Competing interests

All authors declare no competing interests.

## References

[1] Bruni L, Barrionuevo-Rosas L, Albero G, Serrano B, Mena M, Gómez D, Muñoz J, Bosch FX, de Sanjosé S (2016). ICO Information Centre on HPV and Cancer (HPV Information Centre) Human Papillomavirus and Related Diseases in the World..

[2] World Health Organization (2012). International agency for research in cancer.

[3] Mosha D, Mahande M, Ahaz J, Mosha M, Njau B, Kitali B, Obure J (2009). Factors associated with management of cervical cancer patients at KCMC Hospital, Tanzania: a retrospective cross-sectional study. Tanzania Journal of Health Research..

[4] Forhan SE, Godfrey CC, Watts DH, Langley CL (2015). A systematic review of the effects of visual inspection with acetic acid, cryotherapy and loop electrosurgical excision procedures for cervical dysplasia in HIV-infected women in low-and middle- income countries. J Acquir Immune Defic Syndrome..

[5] World Health Organisation (2012). Globocan.

[6] Centres for disease control and prevention 1993 Revised Classification system for HIV infection and expanded surveillance case definition for AIDS among adolescents and adults. National centre for infectious diseases division of HIV/AIDS..

[7] Ajenifuja KO, Gage JC, Adepiti AC, Wentzensen N, Eklund C, Reilly M, Hutchinson M, Burk RD, Schiffman M (2013). A population based study of visual inspection with acetic acid (VIA) for cervical screening in rural Nigeria. Int J Gynecol Cancer..

[8] Globocan (2012). Cervical Cancer: Estimated Incidence, Mortality and Prevalence Worldwide in 2012. International Agency for Research on Cancer (IARC).

[9] Braaten KP, Laufer MR (2008). Human papillomavirus (HPV), HPV-related disease, and the HPV vaccine. Rev Obstet Gynecol..

[10] World Health Organisation Annual WHO/UNICEF Joint reporting form (July 2015) Geneva Immunisation, Vaccines and Biologicals, World Health Organisation..

[11] African coalition on maternal, newborn and child health (2014). Africa cervical cancer multi indicator incidence and mortality scorecard..

[12] Chokunonga E, Borok MZ, Chirenje ZM, Makunike-Mutasa R, Ndlovu N, Nyakabau AM, Vuma S Zimbabwe National Cancer registry: 2014 Annual Report: pattern of cancer in Zimbabwe..

[13] Information Centre for Cancer Human papillomavirus in Zimbabwe report..

[14] Chin'ombe N, Sebata NL, Ruhanya V, Matarira HT (2014). Human Papillomavirus genotypes in cervical cancer and vaccination challenges in Zimbabwe. Infect Agent Cancer..

[15] UNAIDS United Nations AIDS.

[16] Sachdeva RK, Sharma A, Singh S, Varma S (2016). Spectrum of AIDS defining and non-AIDS defining malignancies in North India. Indian J Med Res..

[17] International Agency for Research on Cancer Information Centre for Human Papillomavirus and cancer..

[18] World Health Organization (WHO) Comprehensive cervical cancer prevention and control: a healthier future for girls and women 2013..

[19] Finocchario-Kessler S, Wexler C, Maloba M, Mabachi N, Ndikum-Moffor F, Bukusi E (2016). Cervical cancer prevention and treatment research in Africa: a systematic review from a public health perspective. BMC Womens Health.

[20] WHO Human Papillomavirus laboratory manual..

[21] Maine D, Hurlburt S, Greeson D (2011). Cervical cancer prevention in the 21st century: Cost is not the only issue. Am J Public Health..

[22] World Health Organisation ICO Information of HPV and Cancer: World..

[23] St Alberts mission hospital St Alberts mission hospital viac annual report..

[24] Hanson CM, Eckert L, Bloem P, Cernuschi T (2015). Gavi HPV programs: Application to implementation. Vaccines (Basel)..

[25] Castellsague X, Paavonen J, Jaisamrarn U, Wheeler CM, Skinner SR (2014). HPV PATRICIA Study Group: risk of first cervical HPV infection and pre-canerous lesions after onset of sexual activity:analysis of women in the control arm of the randomised, control PATRICIA trial. BMC infect Dis..

[26] Collins S, Mazloomzadeh S, Winter H, Blomfield P, Baileyy A, Young LS (2002). High incidence of cervical cancer human papillomavirus infection in women during their first sexual relationship. BJOG Int J Obstet Gynaecol..

[27] Kjaer SK, Chackerlan B, van den Brule AJ, Svare EI, Paull G, Walbomers JM (2001). High-risk human papillomavirus is sexually transmitted:evidence from a follow-up study of virgins starting sexual activity (intercourse). Cancer Epidemiol Biomark Prev Publ Am Assoc Cancer Res Cosponsored Am Soc Prev Oncol..

[28] World Health Organisation Weekly epidemiological record. Relevé épidémiologique hebdomadaire..

[29] Fairley G, Hocking J, Chen M, Donovan, Bradshaw C (2009). Rapid decline in warts after national qudrivalent HPV vaccine program..

[30] Shefer A, Markowitz L, Deeks S, Tam T, Irwin K, Garland SM (2008). Early experience with human papillomavirus vaccine introduction in the United States, Canada and Australia. Vaccine..

[31] Public Health England Human Papillomavirus (HPV) Vaccine coverage in England, 2008/09 to 2013/14: A review of the full six years of the three-dose schedule..

[32] Health Policy Monitor HPV vaccination in Switzerland: where are we?.

[33] Boiron L, Joura E, Largaeron N, Prager B, Uhari M (2016). Estimating the cost-effectiveness profile of a universal vaccination programmee with a nine-valent HPV vaccine in Austria. BMC Infect Dis..

[34] Public Health Association of South Africa (2015). Implementation of HPV vaccination in South Africa..

[35] Grover S, Raesima M, Bvochora-Nsingo M, Chiyapo SP, Balang D, Tapela N, Balogun O, Kayembe MKA, Russell AH, Monare B, Tanyala S, Bhat Jailakshmi B, Thipe K, Mchunga M, Mayisela S, Kiziro B, Ho-Foster A, Gaolebale BE, Baolebale PA, Efstathiou JA, Dryden-Peterson S, Zetola N, Hahn SM, Robertson ESm Lin LL, Morroni C, Ramogola-Masire D (2015). Cervical cancer in Botswana: current state and future steps for screening and treatment programs. Frontiers in Oncology..

[36] Bonanni P, Boccalini S, Bechini A (2009). Efficacy, duration of immunity and cross protection after HPV vaccination: a review of evidence. Vaccine.

[37] Jenkins D (2008). A review of cross-protection against oncogenic HPV by an HPV-16/18 AS04-adjuvanted cervical cancer vaccine: importance of virological and clinical endpoints and implications for mass vaccinations in cervical cancer prevention. Gynaecologic Oncology..

[38] Campos NG, Kim JJ, Castle PE, Ortendahl JD, O'Shea M, Diaz M, Goldie SJ (2012). Health and economic impact of HPV16/18 vaccination and cervical cancer screening in Eastern Africa. Int J Cancer..

[39] Meites E, Kempe A, Markowitz LE (2016). Use of a 2-dose schedule for Human Papillomavirus vaccination- Updated recommendations of the Advisory Committee on Immunization Practices. Morbidity and Mortality Weekly Report..

[40] Paul KT (2016). 'Saving lives': adapting and adopting Human Papilloma Virus (HPV) vaccination in Austria..

[41] Mangoma JF, Chirenje MZ, Chimbari MJ, Chandiwana SK (2006). An assessment of rural women's knowledge, constraints and perceptions on cervical cancer screening: the case of two districts in Zimbabwe. Afr J Reprod Health..

[42] Song YJ Knowledge, attitude and beliefs in cervical cancer prevention and HPV vaccination among college youths in Taiwan- a gender-based approach..

[43] Brown B, Folayan M (2015). Barriers to the uptake of human papilloma virus vaccine in Nigeria: a population in need. Niger Med J..

[44] Petry KU, Breugelmans JG, Benard S, Lamure E, Littlewood KJ, Hillemanns P (2008). Cost of screening and treatmen of cervical dyskaryosis in Germany. Eur J Gynaecol Oncol..

[45] World Heaith Organisation World Health Organisation Regional Office for Africa: Cervical cancer common amongst African women..

[46] Fallala MS, Mash R (2015). Cervical cancer screening: safety, acceptability and feasibility of a single-visit approach in Bulawayo, Zimbabwe. Afr J Prm Health Care Fam Med..

[47] Chirenje ZM, Rusakaniko S, Kirumbi L, Ngwalle EW, Makuta-Tlebere PP, Kaggwa S, Mpanju-Shumbusho W, Makoae L (2001). Situation analysis for cervical cancer diagnosis and treatment in East, Central and Southern African countries. World Health Organisation..

[48] Eke NO, Eke CO, Nwosu BO, Akabuike JC, Ezeigwe CO (2006). Okoye SC Cervical cancer screening by female workers in South East Nigeria. African Journals Online..

[49] Albrow R, Kitchener H, Gupta N, Desai M (2012). Cervical screening in England: the past, present and future. Cancer Cytopathology..

[50] Thistle PJ, Chirenje Z M (1997). Cervical cancer screening in rural population of Zimbabwe. Centr Afr J Med..

[51] Chirenje ZM, Akino V (1993). Screening for cervical cancer: experience from the Colposcopy Clinic at Harare Hospital. Cent Afr J Med..

[52] Mafuva C, Djarova T, Matarira HT (2002). Influence of combined oral contraceptives on the onsent of cervical intraepithelial neoplasia. Afr J Health Sci..

[53] Rosser JI, Hamisi S, Njoroge B, Huchko MJ (2015). Barriers to cervical cancer screening in rural Kenya: perspectives from a provoder survey. J Community Health..

[54] Moyo IM, Koni NP, Makunike B, Hipshman J, Makaure HK, Gumbo N (1997). Evaluation of cervical cancer screening programme in the Harare City Health Department, Zimbabwe. Centr Afr J Med..

[55] Mbizvo EM, Msuya SE, Stray-Pedersen B, Chirenje MZ, Hussain A (2005). Cervical dyskaryosis among women with and without HIV: prevalence and risk factors. Int J of STDs and AIDS..

[56] Abotchie PN, Shokar NK (2010). Cervical cancer screening among college students in Ghana: knowledge and health beliefs. Int J Gynaecol Cancer..

[57] CDC STD treatment guidelines..

[58] Sellors J, Camacho Carr K, Bingham A, Winkler J (2004). Course in Visual Methods for Cervical Cancer Screening: visual Inspection With Acetic Acid and Lugol's Iodine..

[59] Spitzer M (1999). Lower genital tract intraepithelial neoplasia in HIV-infected women: guidelines for evaluation and management. Obstet Gynecol Surv..

[60] World Health Organization World Health Organization guidance note: comprehensive cervical cancer prevention and control: a healthier future for girls and women..

[61] Kitchener HC, Syonds P (1999). Detection of cervical intraepithelial neoplasia in developing countries. Lancet..

[62] Sherigar B, Dalal A, Dardi G, Pujar Y, Dhumale H (2010). Cervical cancer screening by visual inspection with acetic-interobserver variability between nurse and physician. Asian Pac J Cancer Prev..

[63] Denny L, Kuhn L, Hu CC, Tsai WY, Wright TC (2010). Human papillomavirus-based cervical cancer prevention: long-term results of a randomized screening trial. J Natl Cancer Inst..

[64] Sankaranarayanan R, Basu P, Wesley RS, Mahe C, Keita N, Mbalawa CC, Parkin DM (2004). Accuracy of visual screening for cervical neoplasia: results from an IARC multicentre study in India and Africa. Multicenter Study Int J Cancer..

[65] Saslow D, Solomon D, Lawson HW, Killackey M, Kulasingam SL, Cain J, Myers ER (2012). American Cancer Society, American Society for Colposcopy and Cervical Pathology, and American Society for Clinical Pathology screening guidelines for the prevention and early detection of cervical cancer. Practice Guideline Am J Clin Pathol..

[66] Cervical cancer prevention fact sheet Ten key findings and recommendations for effective cervical cancer screening and treatment programs..

[67] Bobdey S, Sathwara J, Jain A, Balasubramaniam G (2016). Burden of cervical cancer and role of screening in India. Indian J Med Paediatr Oncol..

[68] International Federation of Gynaecology and Obstetrics Global guidance for cervical cancer prevention and control..

[69] Singla S, Mathur S, Kriplani A, Agarwal N, Garg P, Bhatla N (2012). Single visit approach for management of cervical intraepithelial neoplasia by visual inspection & loop electrosurgical excision procedure. Indian J Med Res..

[70] Chumworathayi B, Blumenthal PD, Limpaphayom KK, Kamsa-Ard S, Wongsena M, Supaatakorn P (2010). Effect of single-visit VIA and cryotherapy cervical cancer prevention program in Roi Et, Thailand: a preliminary report. J Obstet Gynaecol Res..

[71] Khuhaprema T, Attasara P, Srivatanakul P, Sangrajrang S, Muwonge R, Sauvaget C, Sankaranarayanan R (2012). Organization and evolution of organized cervical cytology screening in Thailand. Int J Gynaecol Obstet..

[72] Lazcano-Ponce E, Lörincz AT, Salmerón J, Fernández I, Cruz A, Hernández P (2010). A pilot study of HPV DNA and cytology testing in 50,159 women in the routine Mexican Social Security Program. Cancer Causes Control..

[73] Sankaranarayanan R (2012). 'See-and-treat' works for cervical cancer prevention: what about controlling the high burden in India. Int J Med Res..

[74] Hasahya OT, Berggren V, Sematimba D, Nabirye RC, Kumakech E (2016). Beliefs, perceptions and health seeking behavious in relation to cervical cancer: a qualitative study among women in Uganda following completion of an HPV vaccination campaign. Global Health Action..

[75] Chirenje ZM, Rusakaniko S, Kirumbi L, Ngwalle EW, Makuta-Tlebere P, Kaggwa S, Mpanju-Shumbuso W, Makoae L (2001). Situation analysis for cervical cancer diagnosis and treatment in east, central and southern African countries. Bull World Health Organ..

[76] Denny L, Kuhn L, Hu CC, Tsai WY, Wright TC (2010). Human papillomavirus-based cervical cancer prevention: long-term results of a randomized screening trial. J Natl Cancer Inst..

[77] Chirenje ZM, Rusakaniko S, Akino V, Munjoma M, Mlingo M (2003). Effect of HIV disease in treatment outcome of cervical squamous intraepithelial lesions among Zimbabwean women. J Low Genit Tract Dis..

[78] Cho H, Kim JH (2009). Treatment of the patients with abnormal cervical cytology: a 'see-and-treat' versus three-step strategy. J Gynecol Oncol..

[79] Chirenje ZM, Rusakaniko S, Akino V, Mlingo M (2000). A review of cervical cancer patients presenting in Harare and Parirenyatwa Hospitals in 1998. Centr Afr J Med..

[80] Ndlovu N, Kambarami R (2003). Factors associated with tumour stage at presentation in invasive cervical cancer. Centr Afr J Med..

[81] Texas Oncology cancer.

[82] Kadzatsa W, Chokunonga E The Status and Challenges of Cancer Care in Zimbabwe..

[83] Khumalo T, Maasdorp V (2016). The island hospice model of palliative care. eCancer Medical Science..

